# High rates of loss of heterozygosity on chromosome 19p13 in human breast cancer

**DOI:** 10.1054/bjoc.2000.1606

**Published:** 2001-02

**Authors:** S Oesterreich, D C Allredl, S K Mohsin, Q Zhang, H Wong, A V Lee, C K Osborne, P O'Connell

**Affiliations:** Department of Medicine and Department of Molecular and Cellular Biology; Department of Pathology, Baylor College of Medicine, One Baylor Plaza, Houston, TX, 77030

**Keywords:** loss of heterozygosity, chromosome 19p13, breast cancer, SAFB, tumour suppressor gene, mutation

## Abstract

We have recently discovered that the nuclear matrix protein SAFB is an oestrogen receptor corepressor. Since it has become clear that many steroid receptor cofactors play important roles in breast tumorigenesis, we investigated whether SAFB could also be involved in breast cancer. To address this question, the gene locus was examined for structural alterations in breast cancer tissue. Laser capture microdissection was used for isolating DNA from paired primary breast tumour and normal tissue specimens, and the loss of heterozygosity (LOH) at chromosome 19p13.2–3 was determined by use of microsatellite markers. LOH was detected at the marker D19S216, which colocalizes with the SAFB locus, in specimens from 29 (78.4%) of 37 informative patients. The peak LOH rate occurred at D19S216 near the SAFB locus, with LOH frequencies ranging from 21.6% to 47.2% at other markers. The finding of a very high LOH rate at the marker D19S216 strongly indicates the presence of a breast tumour-suppressor gene locus. While preliminary findings of mutations in SAFB suggest that this indeed may be a promising candidate, other potential candidate genes are located at this locus. © 2001 Cancer Research Campaign http://www.bjcancer.com


British Journal of Cancer (2001) 84(4), 493–498
© 2001 Cancer Research Campaign
doi: 10.1054/ bjoc.2000.1606, available online at http://www.idealibrary.com on IDEAL®

### High rates of loss of heterozygosity on chromosome 19p13 in human breast cancer

**S Oesterreich[1], DC Allredl[1,2], SK Mohsin[1,2], Q Zhang[1], H Wong[1], AV Lee[1], CK Osborne[1] and P O'Connell[1]**

[1]Breast Center, Department of Medicine, and Department of Molecular and Cellular Biology; [2]Department of Pathology, Baylor College of Medicine, One Baylor Plaza, Houston, TX 77030

**Summary** We have recently discovered that the nuclear matrix protein SAFB is an oestrogen receptor corepressor. Since it has become clear that many steroid receptor cofactors play important roles in breast tumorigenesis, we investigated whether SAFB could also be involved in breast cancer. To address this question, the gene locus was examined for structural alterations in breast cancer tissue. Laser capture microdissection was used for isolating DNA from paired primary breast tumour and normal tissue specimens, and the loss of heterozygosity (LOH) at chromosome 19p13.2–3 was determined by use of microsatellite markers. LOH was detected at the marker D19S216, which colocalizes with the SAFB locus, in specimens from 29 (78.4%) of 37 informative patients. The peak LOH rate occurred at D19S216 near the SAFB locus, with LOH frequencies ranging from 21.6% to 47.2% at other markers. The finding of a very high LOH rate at the marker D19S216 strongly indicates the presence of a breast tumour-suppressor gene locus. While preliminary findings of mutations in SAFB suggest that this indeed may be a promising candidate, other potential candidate genes are located at this locus. © 2001 Cancer Research Campaign http://www.bjcancer.com

**Keywords**: loss of heterozygosity; chromosome 19p13; breast cancer; SAFB; tumour suppressor gene; mutation

The oestrogen receptor (ER) is a nuclear steroid receptor that upon activation by its ligands (e.g. oestrogen) initiates a cascade of events resulting in increased cellular proliferation in its target tissues (Warner et al, 1999). Since oestrogen is one of the most potent mitogens for breast cancer cells, it is no surprise that ER is the most important target for endocrine therapy of breast cancer (Osborne, 1998). Recently, a number of factors which regulate nuclear hormone receptor activity have been identified. Cofactors capable of increasing receptor action, termed coactivators, include transcriptional intermediary factor 1 (TIF1), nuclear receptor interacting protein (NRIP1), nuclear receptor coactivator 2 (TIF2), steroid receptor coactivator 1 (SRC1), amplified in breast cancer 1 (AIB1), the cyclic AMP (cAMP)-response element binding protein (CREB) binding protein (CBP) (Glass et al, 1997; Shibata et al, 1997) and many more. The family of corepressors (negative regulators) of ER is smaller; the best characterized ones being the nuclear receptor corepressor (N-CoR) (Horlein et al, 1995; Shibata et al, 1997) the silencing mediator of retinoid and thyroid receptors (SMRT) (Chen and Evans, 1995; Sande and Privalsky, 1996) and the repressor of ER activity (REA) (Montano et al, 1999). The overexpression of coactivators or the loss of corepressors could lead to deregulation of oestrogen-dependent pathways related to mammary epithelial cell proliferation, and thus to breast tumorigenesis. And indeed, some of the ER cofactors have recently been characterized as playing major roles in breast tumorigenesis (Horlein et al, 1995; Anzick et al, 1997; Shibata et al, 1997). The ER coactivator AIB1 was cloned during a search on the long arm of chromosome 20 for genes whose expression and copy number are elevated in human breast cancer, and subsequent analysis in 105 breast tumour specimens confirmed its overexpression (Anzick et al, 1997). Interestingly, the breast/ovarian tumour suppressor gene *BRCA1* has recently been characterized as an ER corepressor (Fan, 1999) again suggesting that ER coregulators are crucial in breast tumorigenesis. Thus, it might be expected that other ER coactivators and corepressors might play similar important roles in breast cancer development and progression.

The nuclear matrix protein SAFB (Renz, 1996; Oesterreich, 1997) has been shown to be an ER corepressor (Oesterreich et al, 2000). ER and SAFB interact in in-vitro binding assays (Glutathione-S-Transferase [GST]-pulldown assays) and in cell lines (co-immunoprecipitation experiments). In cell lines, there is binding of SAFB to ER in the presence or absence of oestradiol; however, binding is significantly increased by the antioestrogen tamoxifen. Overexpression of SAFB results in repression of oestrogen-mediated transactivation of gene expression by the ER. Furthermore, as a result of SAFB overexpression, the antagonist activity of tamoxifen on ER can be enhanced, and the agonist activity of tamoxifen can be inhibited.

These results led us to investigate whether the ER corepressor SAFB could also be involved in breast tumorigenesis. Towards this goal we analysed whether the chromosomal locus for SAFB is a frequent target for chromosomal aberrations, i.e., allelic deletion. Allelic deletion manifested as loss of heterozygosity (LOH) at polymorphic loci is recognized as a hallmark for genes involved in tumour suppression; thus, high LOH at the SAFB locus would suggest that this recently identified ER cofactor could play an important role in breast tumour suppression. In the present study we proposed to study human breast cancer specimens for the rate of LOH at different markers that colocalize with or are adjacent to the SAFB locus on chromosome 19p13. To strengthen our hypothesis we also performed mutational analysis of SAFB in both LOH-positive tumours as well as in breast cancer cell lines.

*Received 26 June 2000*
*Revised 23 October 2000*
*Accepted 8 November 2000*

*Correspondence to:* P O'Connell

### METHODS

### Patients, tissues, and microdissection

The 57 patients whose tissue was evaluated in this study had primary breast cancer; their archival paraffin-embedded tissues were used for the analysis. For 52 of the 57 patients, a single paraffin section yielded sufficient normal tissue (terminal duct lobular unit) and primary cancer. For 5 patients, normal lymph node tissues were recovered from separate blocks. Single 5 μm sections were cut from the selected blocks, mounted on glass slides, deparaffinized, and lightly counterstained with nuclear fast red to guide laser capture microdissection (LCM) of cells using an LCM instrument (Pixcell by Arcturus Engineering) (Emmert-Buck et al, 1996; Simone et al, 1998). Briefly, a transparent thermoplastic film (ethylene vinyl acetate polymer) was placed over the section on the slides. A laser directed through the microscope optics was activated, causing the thermoplastic film to melt and fuse with the underlying targeted cells. The selected cells remained adherent to the film when it was removed from the slide. An average of approximately 1000 cells (about 100 cell clusters of 10 cells each) was harvested from each tissue sample.

### LOH analysis

LOH analysis was performed as recently described (O'Connel et al, 1999). Briefly, DNA was prepared by a modification of the method of Wright and Manos (Wright and Manos, 1990). The embedded cells were incubated for 18–20 hours at 37˚C in 60 μl of a lysis buffer that contained 10 mM Tris-HCl (pH 8.5), 1 mM EDTA, 0.045% NP-40, 0.045% Tween-20, and 1.0 mg $ml^{-1}$ proteinase K. The proteinase was then inactivated at 95˚C for 10 minutes. PCR and gel electrophoresis was performed as described previously by us (O'Connell et al, 1999). Samples were evaluated for LOH using the microsatellite markers D19S216, D19S413, D19S591 and D19S883. The primer pairs were obtained from Research Genetics, Inc. (Birmingham, AL). Mapping data were obtained from the Genome DataBase (GDBTM) at Johns Hopkins University (Fasman et al, 1997; Talbot and Cutichia, 1999). The intensity ratios of bands in electrophoretic gels representing different marker alleles in the DNA obtained from paired normal and breast cancer tissues were calculated from digitized data collected with a storage phosphor device and analysed with the Molecular Dynamics ImageQuant software package (Molecular Dynamics, Sunnyvale, CA). LOH was considered positive when the proportion ((tumour allele 1/tumour allele 2)/(normal allele 1/normal allele 2)) equalled either less than 0.71 (tumour allele 1 LOH) or greater than 1.4 (tumour allele 2 LOH).

### Mutational analysis

RNA from MCF-7/MG (Oesterreich et al, 1993), T47D, and MDA-MB-468 breast cancer cell lines was isolated using Qiagen RNeasy kit (Valencia, CA) according to the manufacturer's instructions. First strand SAFB cDNAs were synthesized in two parts (a 5′ and 3′ segment) by reverse transcriptase (RT)-PCR using Avian Myeoblastosis Virus (AMV) RT (Promega, Madison, WI) on 1 μg of total RNA as previously described (Wang et al, 1999). The primers for RT were 5′-GAGTCTCTTGACTTCC-GAGGC-3′ (for 5′ fragment) and 5′-TCCAAGTACTCAG-TAGCGGCG-3′ (for 3′ fragment). Multiple PCR primers were designed to amplify overlapping regions covering the total cDNA (see footnotes for Table 2). The amplified PCR products were cloned using a TA cloning kit (Invitrogen, Carlsbad, CA), and DNA was isolated and sequenced from at least two clones using Quiaprep Miniprep kit (Quiagen, Valencia, CA).

To analyse the genomic DNA from the LOH-positive tumours, we have started mapping the exon/intron structure of the human SAFB gene by PCR and sequencing. So far we have identified 10 exons, and we designed primer pairs to partially amplify three exons (1F: 5′ATGGCGAGAGGACGGACT-3′ and 1R (intronic): 5′-gcgtctggtctaaaactgagaa-3′, product size = 271 bp; QP1F: 5′-GACTCTGTCAGGCCTAGGTGATTC-3′ and QP1R: 5′-GCTTCATCCAACACACTGATATCC-3′, product size 401 bp; QP6F: 5′-GAGCTTCCAAAAGCCAGGATCGC-3′ and QP6R: 5′-CGCTCCTGCTCATAGCGCAGTT-3′, product size = 364 bp). We analysed 11 tumours with 1F/R, 15 tumours with QP1F/R, and 2 tumours with QP6F/R. The PCR products were cleaned using Quiaquick PCR purification kit (Quiagen), and directly sequenced. The PCR was performed twice, and the product sequenced from both orientations.

### Sequencing

The sequence of cDNA was determined using an Applied Biosystems model 310 genetic analyser.

### Statistical analysis

The confidence intervals were calculated with the expression 1.96, $\sqrt{P \times (1–P)/i}$ where $p$ = the LOH frequency and $i$ = number of informative patients (Dawson Saunders and Trapp, 1994).

### RESULTS

We had previously assigned SAFB to chromosome 19, band p13.2–13.3 by fluorescent in situ hybridization (DuPont et al, 1997). This assigment was subsequently confirmed on the chromosome 19 radiation hybrid map (Deloukas et al, 1998), where SAFB is positioned at 34.7 cRays. As can be seen in Table 1, additional markers and genes have been positioned on chromosome 19p by a combination of FISH, genetic linkage mapping (in centimorgans or cM), and/or radiation hybrid mapping (in centirays or cR). Based on the genetic and physical mapping of this region, 1 cM approximates 120 kilobasepairs (kb) and 1 cR approximates 90 kb on this map. The polymorphic marker D19S216 has been placed on both maps (20.1 cM, 35.9 cRays), so that HET-SAF-B maps in the D19S591-D19S216 interval just proximal to D19S216. This region was tested for LOH using D19S216 and a series of additional markers spanning the chromosome band 19p13, namely, D19S883 (5.5 cM), D19S591 (9.8 cM), D19S216 (20.1 cM), and D19S413 (31.3 cM); which span 3.4 megabasepairs of DNA (Table 1). LOH studies were carried out by comparison of normal and primary breast cancer tissues from 57 patients. 3 of the specimens showed evidence of microsatellite instability and were excluded from further analysis. The results of this LOH study are shown in Table 2. Marker D19S216 near SAFB showed the highest rate of LOH (78%). Figure 1A shows a representative example of an LOH, and Figure 1B summarizes the data from the subset of 25 D19S216-informative patients with interstitial LOH events. An additional 12 patients (not presented) either showed no LOH, or showed LOH for all markers. These breakpoints can map

© 2001 Cancer Research Campaign

**Table 1** Markers and breast cancer candidate genes in 19p13.3

| Marker[a] | Description | Genetic distance (cM)[b] | RH distance (cR)[c] | Megabases[d] |
|---|---|---|---|---|
| APCL | Adenomatous polyposis coli-like | – | 6.1 | 16.6+/– 4.4 |
| D19S883 | Microsatellite AFMa299yc1 | 5.5 | 22[a] | 15.5+/– 0.0 |
| GADD45B | Growth arrest- and DNA damage-inducible gene GADD45, beta | – | 23.4 | – |
| D19S591 | Microsatellite CHLC.GATA44F10 | 9.84 | 27[e] | 15.9+/– 0.0 |
| CDC34 | Cell division cycle 34 | – | – | 16.6+/– 4.4 |
| SH3GL1 | SH3domain, GRB2-like 1 | – | 32.74 | 16.6+/– 4.4 |
| RANB3 | RAN-binding protein 3 | – | 33.94 | 16.6+/– 4.4 |
| SAFB | Scaffold attachment factor B | – | 34.66 | 19.4+/– 7.2 |
| D19S216 | Microsatellite AFM164zb8 | 20.01 | 35.88 | 16.7+/–0.0 |
| TRIP10 | Thyroid hormone receptor interactor 10 | – | 40.66 | – |
| INSR | Insulin receptor | 25.17 | 41.55 | 17.8 +/–0.0 |
| D19S413 | Microsatellite AFM292wd9 | 32.39 | 59.76 | 18.9+/–0.0 |

[a]Genome Database nomenclature (Talbot and Cuticchia, 1999). [b]Distances in centimorgans (cM) from the Marshfield Chromosome 19 Sex-Averaged linkage map (Broman et al, 1998). [c]Distances in centirays (cR) from the International Radiation Hybrid Mapping Consortium (GeneMap'99) (Deloukas et al, 1998). [d]Distances from the Genome Database (Talbot and Cuticchia, 1999). [e]RH distances inferred based on International Radiation Hybrid Mapping Consortium (GeneMap'99) (Deloukas et al, 1998).

**Table 2** Loss of heterozygosity[a] frequencies for genetic markers on chromosome 19p13.3 in breast cancer patients

| Marker | Location (centimorgans) | LOH frequency = no. of patients with LOH / no. of informative patients (%); (95% confidence interval)[b] |
|---|---|---|
| D19S883 | 5.5 | 8/37 (21.6); (8.3–34.9) |
| D19S591 | 9.8 | 17/36 (47.2); (30.9–63.5) |
| D19S216 | 20.1 | 29/37 (78.4); (65.1–91.7) |
| D19S413 | 31.2 | 11/35 (31.4); (16.0–46.8) |

Heterozygosity is the presence of two different alleles for the genetic marker; loss of heterozygosity (LOH) is present when tumour/normal allele intensities calculated as below vary from those seen in normal tissue: LOH is present when ((tumour allele 1/tumour allele 2)/(normal allele 1/normal allele 2)) ratio is either equal to or less than 0.71 for tumour allele 1 or is equal to or greater than 1.4 for tumour allele 2. bThe number of LOH events observed divided by the subset of those patients out of the 54 tested whose normal DNA sample was heterozygous for the genetic marker (informative cases). LOH events cannot be detected in a patient whose normal DNA is homozygous for the genetic marker tested. The confidence interval is calculated with the expression $1.96\sqrt{P \times (1-P)/i}$, where p = the LOH frequency and i = number of informative patients(19).

the smallest region of overlap for the LOH region(s). The majority of the patients show LOH events in the 3 megabasepair region spanning D19S591–D19S216. 4 patients (numbers 96, 179, 1086 and 1094) showed LOH events but remained heterozygous for D19S216, indicating the tumour suppressor is distal of D19S216 (i.e, near SAFB). LOH events in 4 other patients (numbers 190, 207, 613 and 742) lost only D19S216, and patient 810 lost DNA sequences including D19S216 and D19S413. No D19S216-informative tumours exclusively lost D19S413. We also did not detect any homozygous deletion. These data suggest that the interval between D19S591–D19S216 including SAFB harbours a tumour suppressor gene important in human breast cancer. To support our hypothesis, mutational analysis of the remaining SAFB allele was performed in the LOH-positive tumours. SAFB cDNA was also sequenced in 3 breast cancer cell lines.

First we analysed transcripts from MCF-7/MG, T47D and MDA-MB-468 breast cancer cell lines. RT-PCR amplification followed by subcloning of the PCR product and sequencing led to the identification of 3 point mutations changing amino acids (Table 3). The presence of these mutations was confirmed by direct sequencing of genomic DNA from the cell lines (data not shown). To further search for mutations, we PCR-amplified genomic DNA from the nondeleted allele in the LOH-positive tumours, and the results of this study are shown in Table 3. Two point mutations were identified which resulted in amino acid changes, and which were not detected in the adjacent normal tissue. Thus, evidence from sequence analysis of SAFB suggests that the gene indeed is a promising candidate for a breast cancer tumour suppressor gene at the high LOH locus on chromosome 19p13.

### DISCUSSION

Several groups have performed LOH studies on chromosome 19p13. Kerangueven et al [Kerangueven, 1997] identified D19S216 as a marker with consistent loss (20–30% of patients) in breast cancer using genomic DNA isolated from whole breast tumours. Bignell et al (1998) also performed an LOH study on chromosome 19p13.3, with the goal of analysing chromosomal loss of the LKB1 gene (the serine/threonine kinase LKB1 is mutated in patients with Peutz-Jeghers Syndrome, resulting in intestinal hamartomas associated with an elevated risk for cancer). They used the LKB-linked marker D19S565, which co-localizes with D19S883. The Bignell study detected LOH in 7.5% of informative breast cancer specimens, as compared with 21.6% in our study.

It is difficult to compare the LOH rates from our present LCM-based study to those of previous reports, since only a few studies using LCM material have been published. For instance Bignell et al saw 7.5% (3 of 40) LOH with D19S565 using whole tissue genomic DNA while we found 21.6% (8 of 37) using LCM material. Though part of this difference might simply reflect the small number of samples, we have previously seen that LCM enriches for tumour cells and thus always results in a higher LOH rate. As an example, we found 53% LOH (32 of 60) at D19S216 using an essentially identical set of manually microdissected archival paraffin-embedded primary breast cancer specimens (data not shown), but saw 78% LOH (29 of 37) using LCM. Brown et al (1999) also noted elevated LOH rates at 8p12–22 in ovarian

*© 2001 Cancer Research Campaign* 

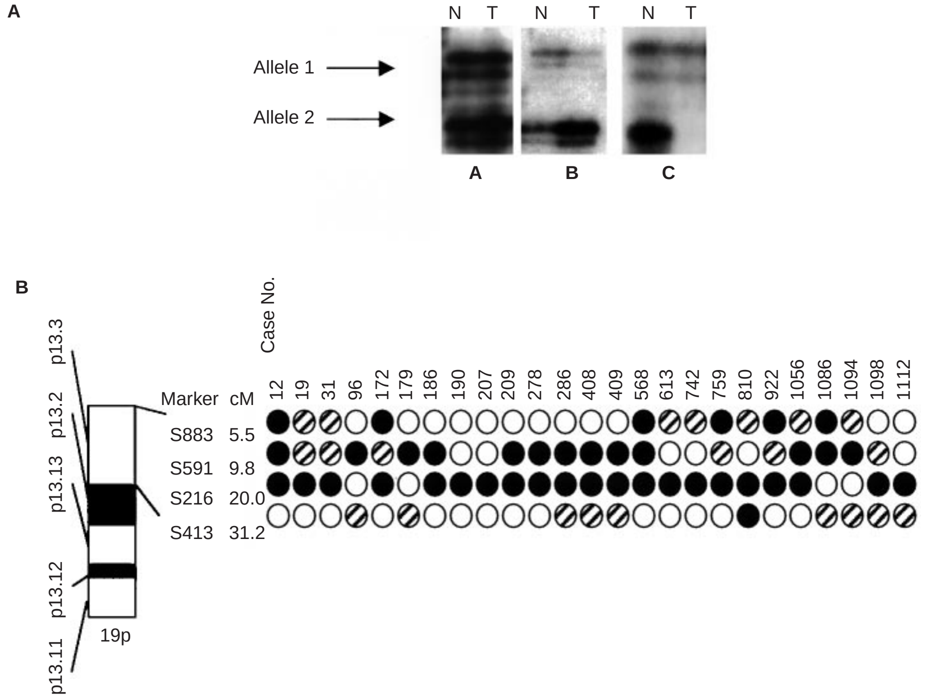

**Figure 1** Loss of heterozygosity (LOH) profiles in the D19S216-SAFB region. (**A**) DNA from microdissected tumour samples (T) and normal (N) corresponding material was analysed by PCR using the microsatellite marker D19S216. Left: no LOH, Middle: LOH – loss of allele 1; right: LOH – loss of allele 2. (**B**) Bottom: An idiogram of chromosome 19p13.11–p13.3 detailing the region of interest and the locations of the markers tested in centimorgans (cM). Top: The LOH profiles of 25 selected patients (patient numbers: 12 to 1112) informative for D19S216 with interstitial breakpoints. Data for breast cancer patient numbers 12–1112 are shown horizontally for each marker. Filled circles denote patients with LOH, open circles denote heterozygous patients (no LOH), and hatched circles show non-informative patients cancers when comparing LCM-based LOH rates with those determined in previous allelotyping studies. Tamura et al (1994) noted 35% LOH at the retinoblastoma (RB) locus on chromosome 13 from whole tumours, but a 59% rate of RB locus LOH when the tumour cells were enriched by flow sorting. We have also determined a rate of 56% LOH at the RB locus (data not shown) in our LCM-based breast cancer studies.

Our rationale for this study was that the ER corepressor SAFB might represent a new tumour suppressor gene, and our present finding would certainly support this hypothesis. LOH frequency at D19S591–SAFB–D19S216 region is among the highest yet measured in breast cancer, and mutational analysis of the SAFB gene in both human breast cancer cell lines and tumours revealed point mutations resulting in amino acid changes. Our sequence analysis so far examined approximately 13% of the SAFB exon sequence from 28 tumours. Although further sequence analysis might lead to the identification of additional mutations in those tumours, our preliminary results indicate that the mutation rate is not very high. It is possible that other epigenetic changes might play a role in inactivating SAFB. Inactivations of tumour suppressor genes through methylation (Merlo et al, 1995; Esteller et al, 2000; Simpson et al, 2000), through altered ubiquitin degradation (Pagano et al, 1995; Tam et al, 1997; Scheffner, 1998; Zaika et al, 1999), and through mislocalization (Chen et al, 1995) are increasingly recognized as alternative inactivating mechanisms. Our own Western blot analyses have demonstrated variations in the abundance of SAFB in breast tumour specimens – in 16% of the tumours (10/61), no SAFB protein was detectable even after prolonged exposure of X-ray films, and in an additional 3% (2/61), SAFB appeared to be truncated (Townson et al, 2000). Thus, other inactivating mechanisms might indeed be involved in loss of SAFB. Recent studies have suggested that haploinsufficiency of some tumour suppressor genes is sufficient for tumorigenesis (Kairouz et al, 1999; Cook and McCaw, 2000)

Despite these observations, we can not exclude that another gene in close proximity to SAFB functions as a tumour suppressor gene in human breast cancer. The 19p13 region studied spans approximately 3 megabasepairs of DNA (see Table 1). A total of 20 known genes and 88 ESTs have been placed in this region

**Table 3** SAFB cDNA and genomic DNA mutations in breast cancer cell lines and LOH-positive tumours

| Cell line/Case[a] | Codon | Nucleotide change | Amino acid change |
|---|---|---|---|
| MCF-7/MG[b] | 1891 | AAG/AGG | Lys/Arg |
| T47D[c] | 1391 | CTC/CCC | Leu/Pro |
| MDA-MB-468[d] | 265 | AAT/GAT | Asn/Asp |
| Tumor #48[e] | 1186 | GCT/GTT | Ala/Val |
| Tumour #30[e] | 1838 | GCC/GGC | Ala/Gly |

[a]The complete cDNA from 3 breast cancer cell lines was analysed. The sequence analysis of the normal/tumour DNA covers 10%, 14% and 13% of the SAFB exon sequence from 11, 15 and 2 cases, respectively.
[b]The 3′ first strand cDNA was amplified using forward primer 5’-GGGGTGCC-TGTGATTAGTGT-3′ and reverse primer 5′- TCAGAATGGTAGCGCTCATCC3′.
[c]The 3′ first strand cDNA was amplified using forward primer 5′-TGGACTCTC-TTCTACAACCAGAGC-3′ and reverse primer 5′- GTCACTGTTGCTCGACTT-CTCC-3′. [d]The 5′ first strand cDNA was amplified using forward primer 5′-AATGGCGGAGACTCTGTCAGGC-3′and reverse primer 5′-ACAGGCTGTCTGCCTTGCTC-3′.
[e]For tumours #48 and #30, genomic DNA from microdissected tumour and adjacent normal tissue was amplified using primer pairs 1F/1R and QP6F/6R (see Methods).

 © 2001 Cancer Research Campaign by the International Radiation Hybrid Mapping Consortium (GeneMap'99) (Deloukas et al, 1998). In addition to SAFB, 7 other genes in the region are potentially breast cancer related. APCL, a homologue of the APC tumour suppressor gene, maps near D19S883, which is outside the peak region of LOH at D19S216. Furthermore, APCL expression has been reported to be brain-specific (Nakagawa et al, 1999). GADD45B, a homologue of the growth arrest and DNA damage-inducible GADD45 gene (Sheikh et al, 2000), maps near D19S591, 800 kb from the peak of LOH near D19S216. The thyroid hormone interacting protein (TRIP10) (Lee et al, 1995) and the insulin receptor (INSR) (Morris, 1997) gene appear to map distal of D19S216, and in any case these genes seem better candidates as oncogenes rather than tumour suppressor genes. 3 genes, the GRB2-like SH3 domain containing gene (SH3GL1) (Giachino et al, 1997), the Ras-related nuclear protein-binding protein (RANBP3) (Mueller et al, 1998), and $G_2$ cell division cycle checkpoint gene (CDC34) (Kaiser et al, 2000), all map to the same interval as SAFB. However, as noted above, SH3GL1 seems a better candidate for an oncogene than tumour suppressor genes. RANBP3 and CDC34 (or an uncharacterized EST) in this region remain potential candidate tumour suppressor genes in addition to SAFB.

Further studies are necessary to extensively characterize this extremely interesting region on chromosome 19p13, and to firmly establish SAFB as a tumour suppressor gene, or to show that a gene other than SAFB is the true classical tumour suppressor gene at this locus.

### ACKNOWLEDGEMENTS

The authors wish to thank Lei Hao, Toby Sullivan, and Jeffrey Chavez for expert technical assistance, and Gary Chamness for critical reading and Susan Hilsenbeck for helpful discussion of this manuscript. This work was supported by US Department of Defense grant DAMD17–98–8340, and PHS grants K01 CA77654, P50-CA58183 and P01-CA30195.

### REFERENCES

Anzick SL, Kononen J, Walker RL, Azorsa DO, Tanner MM, Guan XY, Sauter G, Kallioniemi OP, Trent JM and Meltzer PS (1997) AIB1, a steroid receptor coactivator amplified in breast and ovarian cancer. *Science* **277**: 965–968

Bignell GR, Barfoot R, Seal S, Collins N, Warren W and Stratton M (1998) Low frequency of somatic mutations in the LKB1/Peutz-Jeghers Syndrome gene in sporadic breast cancer. *Cancer Research* **58** :1384–1386

Broman KW, Murray JC, Sheffield VC, White RL and Weber JL (1998) Comprehensive human genetic maps: individual and sex-specific variation in recombination. *Am J Hum Genet* **63**: 861–869

Brown MR, Chuaqui R, Vocke CD, Berchuck A, Middleton LP, Emmert-Buck MR and Kohn EC (1999) Allelic loss on chromosome arm 8p: analysis of sporadic epithelial ovarian tumors. *Gynecol Oncol* **74**: 98–102

Chen JD and Evans RM (1995) A transcriptional co-repressor that interacts with nuclear hormone receptors. *Nature* **5**: 454–457

Chen Y, Chen CF, Riley DJ, Allred DC, Chen PL, Von Hoff D, Osborne CK and Lee W (1995) Aberrant subcellular localization of BRCA1 in breast cancer. *Science* **270**: 789–791

Cook WD and McCaw BJ (2000) Accommodating haploinsufficient tumor suppressor genes in Knudson's model. *Oncogene* **19**: 3434–3438

Dawson Saunders B and Trapp RG (1994) *Basic and Clinical Biostatistics* 2nd edition. East Norwalk

Deloukas P, Schuler GD, Gyapay G, Beasley EM, Soderlund C, Rodriguez-Tome P, Hui L, Matise TC, McKusick KB, Beckmann JS, Bentolila S, Bihoreau M, Birren BB, Browne J, Butler A, Castle AB, Chiannilkulchai N, Clee C, Day PJ, Dehejia A, Dibling T, Drouot N, Duprat S, Fizames C, Bentley DR and et al. (1998) A physical map of 30,000 human genes. *Science* **282**: 744–746

DuPont BR, Garcia DK, Sullivan TM, Naylor SL and Oesterreich S (1997) Assignment of SAFB encoding Hsp27 ERE-TATA binding protein (HET)/scaffold attachment factor B (SAF-B) to human chromosome 19 band p13. *Cytogenet Cell Genet* **79**: 284–285

Emmert-Buck MR, Bonner RF, Smith PD, Chuaqui RF, Zhuang Z, Goldstein SR, Weiss RA and Liotta LA (1996) Laser capture microdissection [see comments]. *Science* **274**: 998–1001

Esteller M, Silva JM, Dominguez G, Bonilla F, Matias-Guiu X, Lerma E, Bussaglia E, Prat J, Harkes IC, Repasky EA, Gabrielson E, Schutte M, Baylin SB and Herman JG (2000) Promoter hypermethylation and BRCA1 inactivation in sporadic breast and ovarian tumors [In Process Citation]. *J Natl Cancer Inst* **92**: 564–569

Fasman KH, Letovsky SI, Li P, Cottingham RW and Kingsbury DT (1997) The GDB Human Genome Database Anno 1997. *Nucleic Acids Res* **25**: 72–81

Giachino C, Lantelme E, Lanzetti L, Saccone S, Bella Valle G and Migone N (1997) A novel SH3-containing human gene family preferentially expressed in the central nervous system. *Genomics* **41**: 427–434

Glass CK, Rose DW and Rosenfeld MG (1997) Nuclear receptor coactivators. *Current Opinion in Cell Biology* **2**: 222–232

Horlein AJ, Naar AM, Heinzel T, Torchia J, Gloss B, Kurokawa R, Ryan A, Kamei Y, Soderstrom M, Glass CK and et al. (1995) Ligand-independent repression by the thyroid hormone receptor mediated by a nuclear receptor co-repressor. *Nature* **377**: 397–404

Kairouz R, Clarke RA, Marr PJ, Watters D, Lavin MF, Kearsley JH and Lee CS (1999) ATM protein synthesis patterns in sporadic breast cancer. *Mol Pathol* **52**: 252–256

Kaiser P, Flick K, Wittenberg C and Reed SI (2000) Regulation of transcription by ubiquitination without proteolysis: Cdc34/SCF(Met30)-mediated inactivation of the transcription factor Met4. *Cell* **102**: 303–314

Lee JW, Choi HS, Gyuris J, Brent R and Moore DD (1995) Two classes of proteins dependent on either the presence or absence of thyroid hormone for interaction with the thyroid hormone receptor. *Mol Endocrinol* **9**: 243–254

Merlo A, Herman JG, Mao L, Lee DJ, Gabrielson E, Burger PC, Baylin SB and Sidransky D (1995) 5´ CpG island methylation is associated with transcriptional silencing of the tumour suppressor p16/CDKN2/MTS1 in human cancers [see comments]. *Nat Med* **1**: 686–692

Montano MM, Ekena K, Delage-Mourroux R, Chang W, Martini P and Katzenellenbogen BS (1999) An estrogen receptor-selective coregulator that potentiates the effectiveness of antiestrogens and represses the activity of estrogens. *Proc Natl Acad Sci USA* **96**: 6947–6952

Morris BJ (1997) Insulin receptor gene in hypertension. *Clin Exp Hypertens* **19**: 551–565

Mueller L, Cordes VC, Bischoff FR and Ponstingl H (1998). Human RanBP3, a group of nuclear RanGTP binding proteins. *FEBS Lett* **427**: 330–336

Nakagawa H, Koyama K, Monden M and Nakamura Y (1999) Analysis of APCL, a brain-specific adenomatous polyposis coli homologue, for mutations and expression in brain tumors. *Jpn J Cancer Res* **90**: 982–986

O'Connell P, Fischbach K, Hilsenbeck S, Mohsin SK, Fuqua SA, Clark GM, Osborne CK and Allred DC (1999) Loss of heterozygosity at D14S62 and metastatic potential of breast cancer. *J Natl Cancer Inst* **91**: 1391–1397

Oesterreich S, Weng C-N, Qiu M, Hilsenbeck SG, Osborne CK and Fuqua SAW (1993) The small heat shock protein hsp27 is correlated with growth and drug resistance in human breast cancer cell lines. *Cancer Research* **53**: 4443–4448

Oesterreich S, Zhang Q, Hopp T, Fuqua SA, Michaelis M, Zhao HH, Davie JR, Osborne CK and Lee AV (2000) Tamoxifen-bound estrogen receptor (ER) strongly interacts with the nuclear matrix protein HET/SAF-B, a novel inhibitor of ER-mediated transactivation [In Process Citation]. *Mol Endocrinol* **14**: 369–381

Osborne CK (1998) Steroid hormone receptors in breast cancer management. *Breast Cancer Res Treat* **51**: 227–238

Pagano M, Tam SW, Theodoras AM, Beer-Romero P, Del Sal G, Chau V, Yew PR, Draetta GF and Rolfe M (1995) Role of the ubiquitin-proteasome pathway in regulating abundance of the cyclin-dependent kinase inhibitor p27 [see comments]. *Science* **269**: 682–685

Sande S and Privalsky ML (1996) Identification of TRACs (T3 receptor-associating cofactors), a family of cofactors that associate with, and modulate the activity of, nuclear hormone receptors. *Molecular Endocrinology* **10**: 813–825

Scheffner M (1998) Ubiquitin, E6-AP, and their role in p53 inactivation. *Pharmacol Ther* **78**: 129–139

Sheikh MS, Hollander MC and Fornance AJ Jr. (2000) Role of Gadd45 in apoptosis. *Biochem Pharmacol* **59**: 43–45

© 2001 Cancer Research Campaign 

Shibata H, Spencer TE, Onate SA, Jenster G, Tsai SY, Tsai MJ and O'Malley BW (1997) Role of co-activators and co-repressors in the mechanism of steroid/thyroid receptor action. *Recent Progress in Hormone Research* **52**: 141–164

Simone NL, Bonner RF, Gillespie JW, Emmert-Buck MR and Liotta JR (1998) Laser-capture microdissection: opening the microscopic frontier to molecular analysis. *Trends in Genetics* **14**: 272–276

Simpson DJ, Hibberts NA, McNicol AM, Clayton RN and Farrell WE (2000) Loss of pRb expression in pituitary adenomas is associated with methylation of the RB1 CpG island. *Cancer Res* **60**: 1211–1216

Talbot CCJ and Cutichia AJ (1999) Human mapping databases. In: *Current Protocols in Human Genetics*, Vol. 1.13.1–1.13.12. John Wiley & Sons, Inc.

Tam SW, Theodoras AM and Pagano M (1997) Kip1 degradation via the ubiquitin-proteasome pathway. *Leukemia* **11**: 363–366

Tamura G, Maesawa C, Suzuki Y, Kashiwaba M, Ishida M, Saito K and Satodate R (1994) Improved detection of loss of heterozygosity at retinoblastoma gene locus in human breast carcinoma. *Pathol Int* **44**: 34–38

Townson SM, Sullivan T, Zhang Q, Clark GM, Osborne CK, Lee AV and Oesterreich S (2000) HET/SAF-B overexpression causes growth arrest and multinuclearity and is associated with aneuploidy in human breast cancer [In Process Citation]. *Clin Cancer Res* **6**: 3788–3796

Wang Z, Cody JD, Leach RJ and O'Connell P (1999) Gene expression patterns in cell lines from patients with 18q-syndrome. *Hum Genet* **104**: 467–475

Warner M, Nilsson S and Gustafsson JA (1999). The estrogen receptor family. *Curr Opin Obstet Gynecol* **11**: 249–254

Wright DK and Manos MM (1990) Sample preparation from paraffin embedded tissues. In: *PCR protocols*, Innes MA, Gelfand DH and Sninsky JJ (eds) pp. 153–158. Academic Press: San Diego

Zaika A, Marchenko N and Moll UM (1999) Cytoplasmically "sequestered" wild type p53 protein in resistant to Mdm2-mediated degradation. *J Biol Chem* **274**: 27474–27480

*© 2001 Cancer Research Campaign*
